# Human Cellular Immune Response to the Saliva of *Phlebotomus papatasi* Is Mediated by IL-10-Producing CD8+ T Cells and Th1-Polarized CD4+ Lymphocytes

**DOI:** 10.1371/journal.pntd.0001345

**Published:** 2011-10-04

**Authors:** Maha Abdeladhim, Mélika Ben Ahmed, Soumaya Marzouki, Nadia Belhadj Hmida, Thouraya Boussoffara, Nabil Belhaj Hamida, Afif Ben Salah, Hechmi Louzir

**Affiliations:** 1 Department of Clinical Immunology, Pasteur Institute of Tunis, Tunis, Tunisia; 2 LIVGM, Pasteur Institute of Tunis, Tunis, Tunisia; 3 Department of Medical Epidemiology, Pasteur Institute of Tunis, Tunis, Tunisia; Lancaster University, United Kingdom

## Abstract

**Background:**

The saliva of sand flies strongly enhances the infectivity of *Leishmania* in mice. Additionally, pre-exposure to saliva can protect mice from disease progression probably through the induction of a cellular immune response.

**Methodology/Principal Findings:**

We analysed the cellular immune response against the saliva of *Phlebotomus papatasi* in humans and defined the phenotypic characteristics and cytokine production pattern of specific lymphocytes by flow cytometry. Additionally, proliferation and IFN-γ production of activated cells were analysed in magnetically separated CD4+ and CD8+ T cells. A proliferative response of peripheral blood mononuclear cells against the saliva of *Phlebotomus papatasi* was demonstrated in nearly 30% of naturally exposed individuals. Salivary extracts did not induce any secretion of IFN-γ but triggered the production of IL-10 primarily by CD8+ lymphocytes. In magnetically separated lymphocytes, the saliva induced the proliferation of both CD4+ and CD8+ T cells which was further enhanced after IL-10 blockage. Interestingly, when activated CD4+ lymphocytes were separated from CD8+ cells, they produced high amounts of IFN-γ.

**Conclusion:**

Herein, we demonstrated that the overall effect of *Phlebotomus papatasi* saliva was dominated by the activation of IL-10-producing CD8+ cells suggesting a possible detrimental effect of pre-exposure to saliva on human leishmaniasis outcome. However, the activation of Th1 lymphocytes by the saliva provides the rationale to better define the nature of the salivary antigens that could be used for vaccine development.

## Author Summary

Cutaneous leishmaniasis affects millions of people worldwide and is caused by protozoa of the genus *Leishmania*. The parasite is transmitted during sand fly bites. While probing the skin for a blood meal, vectors salivate into the host's skin. Sand fly saliva contains several components that increase hemorrhage and interfere with the host's inflammatory response. Data obtained in mice originally indicate that immunization against saliva protected from leishmaniasis supporting possibility that leishmaniasis could be prevented by a vaccine based on sand fly saliva. Herein we investigated the nature and the importance of the cellular immune response developed against sand fly saliva by individuals at risk of cutaneous leishmaniasis due to *Leishmania major*. We demonstrated that the immunity against saliva is dominated by the activation of lymphocytes producing a suppressive cytokine called IL-10. These data may preclude the protective effect of sand fly saliva pre-exposure in humans. Further experiments revealed that the production of IL-10 masked the presence of a second kind of lymphocytes producing IFN-γ, a rather protective cytokine. The latter finding highlights the importance of the identification of the proteins activating the latter lymphocytes in order to develop vaccines based on selected proteins from the saliva of sand flies.

## Introduction

Leishmaniasis includes a heterogeneous group of diseases that are caused by protozoan parasites of the genus *Leishmania*. The disease ranges from asymptomatic infections to self-limiting cutaneous lesion(s) or fatal visceral forms [1]. *Leishmania* parasites are transmitted to the vertebrate hosts by the bite of sand flies. During parasite inoculation in the host's skin, the vector injects the saliva that contains a large number of pharmacological components [2]–[4]. Several observations indicate that sand fly saliva is crucial in the establishment of leishmaniasis and disease pathogenesis [5]–[7]. The mechanism by which the vector's saliva enhances leishmania infection remains to be clarified. Sand fly saliva contains potent antihemostatic and vasodilatator compounds as well as potentially immunomodulatory molecules that can directly down-modulate macrophage effector functions and facilitate the establishment of the infection [8], [9]. The exacerbating effects of saliva may also be related to the early release of epidermal interleukin-4 (IL-4) [7]. Alternatively, it could be ascribed to the development of an adaptive immune response that would favor the commitment of a Th2 immunity against *Leishmania*. In mice, pre-exposure to saliva completely abrogated the effects of the sand fly saliva and protected the host from disease progression [7], [10], [11]. This protective effect correlated with a strong delayed-type hypersensitivity (DTH) response and an early and increased *in situ* production of IFN-γ and IL-12 [10]. Further experiments demonstrated that immunization with PpSP15 (gi|15963509) from *P. papatasi* saliva resulted in protection which was not ascribed to a humoral immune response [12]. Altogether, these data strongly supported the possibility that leishmaniasis could be prevented by vaccinating against sand fly saliva and suggest that the protective effect of the saliva might be associated with cell-mediated immunity. In humans, the data about the cellular immune responses are scarce. Herein, we analyzed the cellular immune response against the saliva of *Phlebotomus papatasi* developed in individuals naturally exposed to sand fly bites and demonstrated that the overall effect of *Phlebotomus papatasi* saliva was dominated by the activation of peripheral IL-10-producing CD8+ cells. Strikingly, the activation of IFN-γ-producing CD4+ T lymphocytes has been revealed after neutralization of IL-10 production or depletion of CD8 lymphocytes.

## Methods

### Ethic statement

All experiments were conducted according to the principles expressed in the Declaration of Helsinki. The study was approved by the ethic committee of Institute Pasteur of Tunis. All patients provided written informed consent for the collection of samples and subsequent analysis.

### Study population and samples

Peripheral blood samples were drawn from 36 donors **(** **Table 1** **)**. The sampling has been performed on April, just before the transmission season of cutaneous leishmaniasis in Tunisia (between June and October). Ten donors (B1 to B10; age range 27–52 years, mean 31.9 years) were living in Tunis, a non endemic region for ZCL but in which the presence of *P. papatasi* has been reported at low frequency [13]. Twenty-six (B11 to B36; age range 15–73 years, mean 40.2 years) were living in Sidi Bouzid, a region located in the center of Tunisia, which is endemic for ZCL caused by *Leishmania major* and characterized by the presence of *P. papatasi* at high frequencies [13].

**Table 1:** Clinical and biological features of the study population.

Donors	Sex	Age	Scars	Proliferation against SLA(Index of proliferation)	Proliferation against SGE (cpm of stimulated condition/Index of stimulation)	Antibody response(ELISA/Western blot)	LST
**B_1_**	F	28	-	1.22	1628 (0.98)	Neg/Neg	Not Applicable
**B_2_**	F	23	-	**5.49**	**4232 (5.79)**	Neg/**Pos**	Not Applicable
**B_3_**	M	28	-	0.51	1119 (0.77)	Neg/Neg	Not Applicable
**B_4_**	M	27	-	1.20	797 (0.99)	Neg/Neg	Not Applicable
**B_5_**	F	29	-	1.34	499 (0.98)	Neg/Neg	Not Applicable
**B_6_**	M	26	-	0.86	**3786 (2.46)**	**Pos**/**Pos**	Not Applicable
**B_7_**	M	52	-	**12.20**	1627 (0.79)	Neg/Neg	Not Applicable
**B_8_**	F	29	-	1.30	444 (0.99)	Neg/Neg	Not Applicable
**B_9_**	M	47	-	1.12	469 (0.64)	Neg/Neg	Not Applicable
**B_10_**	F	30	-	1.24	455 (0.99)	Neg/Neg	Not Applicable
**B_11_**	F	25	+	**5.68**	**7550 (3.84)**	**Pos/Pos**	Not Applicable
**B_12_**	F	22	-	**27.34**	**5927 (8.83)**	**Pos/Pos**	Not Applicable
**B_13_**	M	52	-	**33.36**	516 (0.62)	**Pos**/**Pos**	Not Applicable
**B_14_**	M	18	-	0.80	378 (0.52)	Neg/Neg	Not Applicable
**B_15_**	M	43	-	**8.81**	930 (0.95)	**Pos/Pos**	**+**
**B_16_**	M	62	-	**14.67**	**4975 (2.99)**	**Pos**/**Pos**	+
**B_17_**	M	70	+	**42.75**	494 (0.82)	Neg/**Pos**	**-**
**B_18_**	F	27	+	**17.76**	681 (0.92)	**Pos/Pos**	**+**
**B_19_**	F	34	+	**29.46**	409 (0.85)	Neg/**Pos**	**+**
**B_20_**	F	19	-	**11.37**	**3861 (2.19)**	**Pos**/**Pos**	-
**B_21_**	F	16	+	**75.04**	368 (0.82)	Neg/**Pos**	+
**B_22_**	F	50	+	**78.17**	**3440 (2.52)**	Neg/**Pos**	+
**B_23_**	F	15	+	**45.60**	372 (0.49)	**Pos/Pos**	+
**B_24_**	F	48	+	**54.39**	360 (0.70)	**Pos/Pos**	+
**B_25_**	F	42	Not Applicable	**9.786**	884 (0.79)	Neg/**Pos**	+
**B_26_**	F	42	-	**32.89**	395 (1.00)	**Pos/Pos**	+
**B_27_**	M	50	+	**13.30**	961 (0.98)	**Pos**/**Pos**	+
**B_28_**	M	33	+	**16.75**	407 (0.73)	**Pos/Pos**	-
**B_29_**	M	43	+	**22.71**	**4093 (2.1)**	Neg/**Pos**	+
**B_30_**	M	48	Not Applicable	1.059	1653 (0.81)	**Pos/Pos**	-
**B_31_**	F	41	Not Applicable	**7.78**	**7980 (4.75)**	**Pos/Pos**	+
**B_32_**	F	49	Not Applicable	**2.79**	191 (0.5)	**Pos/Pos**	+
**B_33_**	F	43	Not Applicable	**2.51**	**3656 (2.19)**	**Pos/Pos**	+
**B_34_**	F	23	Not Applicable	**4.80**	290 (0.51)	Neg**/Pos**	+
**B_35_**	M	38	Not Applicable	**7.74**	**5520 (4.23)**	**Pos/Pos**	+
**B_36_**	M	54	Not Applicable	**23.42**	4107 (0.68)	**Pos/Pos**	+

**M**: Male; **F**: Female; **SLA:** Soluble *Leishmania* antigen**; cpm**: count per minute; **index of stimulation**  =  the ratio of cpm in stimulated condition / unstimulated one

Positive results regarding the cell proliferation against SLA and SGE (index above 2) and the antibody response against SGE are indicated in bold.

### Culture media and reagents

In all *in vitro* assays, cells were cultured in RPMI 1640 medium supplemented with 10% AB human serum (Sigma, St Louis, MO), 1% sodium pyruvate, 1% non essential aminoacids, 1% HEPES buffer, 5×10^−5^ M/L β-mercaptoethanol and 40 µg/mL gentamycin, (Invitrogen, Cergy Pontoise, France).

Purified blocking anti-human IL-10 antibody (BD Biosciences, Le Pont de Claix, France) was used in cell culture. The following monoclonal antibodies were used for flow cytometry analysis: FITC-, and Cy-Chrome-conjugated anti-human CD3, CD4 and CD8, PE-conjugated anti-IL-4, IL-10, IFN-γ, TNF-α, granzyme B and control isotypes (BD Biosciences).

### Salivary gland extract and soluble Leishmania antigen preparation

Salivary glands from a Tunisian strain of *Phlebotomus papatasi* were dissected out in phosphate buffer saline and disrupted by 3 freezing/thawing cycles. After centrifugation, the supernatants were stored at –80°C. Just before use, the salivary gland extract (SGE) was prepared by dilution in cell culture medium added with gentamycin (Invitrogen).

Soluble *Leishmania* antigen (SLA) was prepared from an isolate of *L. major* (zymodme MON25; MHOM/TN/94/GLC94), obtained from ZCL lesion, as previously described [14].

### Lymphocyte isolation and cell culture

Peripheral blood mononuclear cells (PBMC) were isolated on a Ficoll-Hypaque gradient. In some experiments, lymphocyte subsets were separated by negative selection using magnetic beads (Miltenyi Biotec, Paris, France). Purity of T cell subsets ranged from 95% to 98%.

Cells were cultured in 96-well plates in cell culture medium at 0.5×10^6^ cells/mL in a final volume of 200 µL and incubated with SGE (1gland/mL) or SLA (10 µg/mL) with or without anti-IL-10 or isotype control in a 5%CO_2_ humidified atmosphere at 37°C. The optimum condition for PBMC proliferation against SGE was determined in our laboratory in preliminary experiments. We thus stimulated cells with different concentrations of salivary gland extracts (0.25 gland/mL, 0.5 gland/mL, 1 gland/mL and 2 gland/mL) for 3, 4 and 5 days and the optimal results (highest index of proliferation corresponding to the ratio of cpm in stimulated condition / unstimulated one) were obtained with 1 gland/mL during five days. For proliferation studies, the uptake of (^3^H) thymidine (Amersham, Saclay, France) was measured 18 hours after adding 0.4 µCi/well. Cells were harvested and the radioactivity was counted in a scintillation counter (Rack Beta; LKB Wallace). Results were expressed as a proliferation index: mean counts of triplicates in antigen-stimulated cultures /mean counts of triplicates in unstimulated cultures.

### IL-10 and IFN-γ detection assay

For IL-10 or IFN-γ detection, supernatants of cell culture were collected after 48h or 72h, respectively, centrifuged and stored at –80°C until use. Capture enzyme-linked immunosorbent assay (ELISA) was performed on supernatants using Human IL-10 or IFN-γ ELISA Sets (BD Biosciences) according to manufacturer's instructions. For each cytokine determination, the results were interpolated from a standard curve using recombinant cytokines and expressed in pg/mL or as ratio of IFN-γ concentration in stimulated/unstimulated cultures.

### Flow cytometry analysis

Freshly isolated PBMC were stimulated for 24h to 96h with SGE (1gland/mL) or medium alone in 24-well plates and treated with Golgistop (BD Biosciences) for the last 6 hours of culture. Cells were then washed and incubated with FITC or PE-Cy5.5 conjugated to CD3, CD4 or CD8 antibodies for 20 minutes at 4°C. For intracellular cytokine detection, the cells were fixed and permeabilized using BD Cytoperm/cytofix plus kit (BD Biosciences) according to manufacturer's instructions and labeled with PE-conjugated anti-human IFN-γ, IL-4, IL-10, TNF-α, granzyme B or control isotype (BD Biosciences). Analyses were performed with a FACS Vantage flow cytometer using the CELLQuest software (BD Biosciences).

### Serum anti-SGE antibody detection

Specific anti-saliva IgG antibodies were assessed by ELISA and Western blot as previously described [15].

For ELISA, wells were coated with SGE (0.5 gland per well) in 0.1 M carbonate-bicarbonate buffer overnight at 4°C. The wells were then washed in phosphate buffer (PBS) added with 0.1% Tween 20 and incubated with PBS-Tween20–0.5% gelatin for 1 hour at 37°C to block free binding sites. Diluted sera (1∶200) were then incubated for 2 hours at 37°C. Antibody-antigen complexes were detected using peroxidase-conjugated anti-human IgG antibody diluted at 1∶10000 (Sigma) for 1 hour at 37°C and were visualized using Orthophenylendiamine in citrate buffer and hydrogen peroxide. The absorbance was measured using an automated ELISA reader (Awareness Technology Inc) at 492nm wavelength. The cut-off for the assays was the mean optical density obtained with sera of 20 negative controls obtained from the study of Marzouki et al. [15] plus 3 standard deviations.

For Western-blot analysis, the equivalent of 40 to 60 salivary glands were loaded in a single long well and separated on 15% sodium dodecyl sulfate (SDS)-PAGE gel. The separated proteins were then transferred onto a nitrocellulose membrane. The membrane was incubated overnight at 4°C with blocking buffer containing 5% non-fat milk and then cut into 8 to10 strips. Each strip was incubated for one hour at room temperature with diluted serum samples (1∶200). After washing, the strips were incubated with horseradish peroxidase-linked anti-human IgG antibody (Sigma) at 1∶10000 for one hour at room temperature. After five washings, positive bands were visualized using enhanced chemiluminescence (Amersham).

### Statistical analysis

Values obtained in two different groups were compared by the non parametric Mann-Whitney *U* test using StatView software. Statistics of results obtained as paired data were performed using the Paired-T test. The correlation between different parameters was analyzed using Spearman's rank correlation. The exact Fisher test was used to compare the frequency of individuals exhibiting a positive proliferative response against the sand fly saliva between the stratified groups of donors. Statistical significance was assigned to a value of p<0.05.

## Results

### Salivary gland extracts from *P. papatasi* elicit PBMC proliferation in exposed individuals

**Figure 1:**
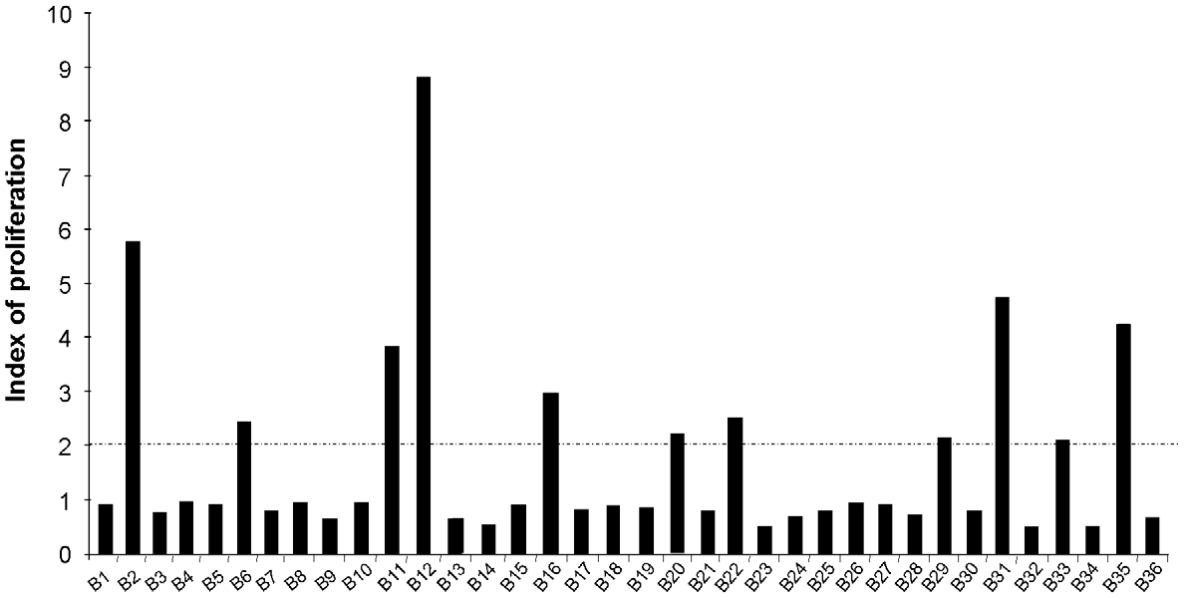
Salivary gland extracts of *Phlebotomus papatasi* induce proliferation of PBMC in previously exposed individuals. Peripheral blood mononuclear cells (PBMC) were isolated from 36 volunteer donors; 10 donors (B1 to B10) were from a non endemic region for ZCL and 26 individuals (B11 to B36) were from endemic areas. PBMC (0.5×10^6^ cells/mL) were stimulated in 96-well plates with salivary gland extracts at one gland/ml during five days. Proliferative responses were assessed by (^3^H) thymidine uptake. Results were expressed as an index of proliferation (mean counts of triplicates in antigen-stimulated cultures /mean counts of triplicates in unstimulated cultures) in all tested individuals. We designated as positive SGE proliferation the cases exhibiting an index of PBMC proliferation above 2. The indices of proliferation obtained in such donors were relatively low with a median of 3.2 but significantly higher than those obtained in donors with negative SGE responses (p<0.0001).

The cellular immune response against *P. papatasi* salivary gland extracts (SGE) was first assessed by studying the proliferative responses of peripheral blood mononuclear cells (PBMC) from our donors. The optimum condition for the proliferation test has been determined previously (namely, stimulation with 1 gland/ml of SGE during five days of culture) **(Figure S1).** We designated as positive SGE proliferation the cases exhibiting an index of PBMC proliferation against the saliva of *P. papatasi* above 2. We thus demonstrated that 11 out of 36 donors exhibited positive SGE proliferation **(** **Figure 1** **)**. The indices of proliferation obtained in these donors were relatively low with a median of 3.2 but significantly higher than those of donors with negative SGE responses (p<0.0001) **(** **Figure 1** **)**. The percentage of individuals with positive SGE proliferation was higher in the group of donors living in endemic area of leishmaniasis than in donors living in northern parts of Tunisia (9 out of 26 donors versus 2 out of 10 donors). The difference was, however, not significant **(p>0.05).**

Twenty-six donors exhibited positive proliferation against SLA (soluble *Leishmania* antigen). Except for B7 who has been accidentally exposed to *Leishmania* parasites while manipulating in a laboratory, the latter results suggest that these donors have been exposed to infected sand fly bites. Consistently, in 18 out of 21 donors with positive proliferation against SLA, a delayed-type hypersensitivity response against *Leishmania* antigens has been demonstrated **(** **Table 1** **).** Interestingly, a cellular immune response against SGE was more frequently noted in individuals who exhibited a PBMC proliferation against SLA compared to those with no proliferation against SLA (10 out of 26 donors versus 1 out of 10 donors) **(** **Table 1** **)**. However, no correlation was found between the level of proliferative responses against SGE and this of proliferation against SLA (soluble *Leishmania* antigen) in all studied donors (p>0.05) **(** **Table 1** **and** **Figure 1** **)**. In donors with a cellular immune response against *L. major* (positive proliferation against SLA), no association between proliferative responses against SGE and the presence or absence of a past history of ZCL was found (p>0.05) **(data not shown)**.

### Cellular immune response against salivary gland extract is dominated by IL-10 and IL-4 secretion

**Figure 2:**
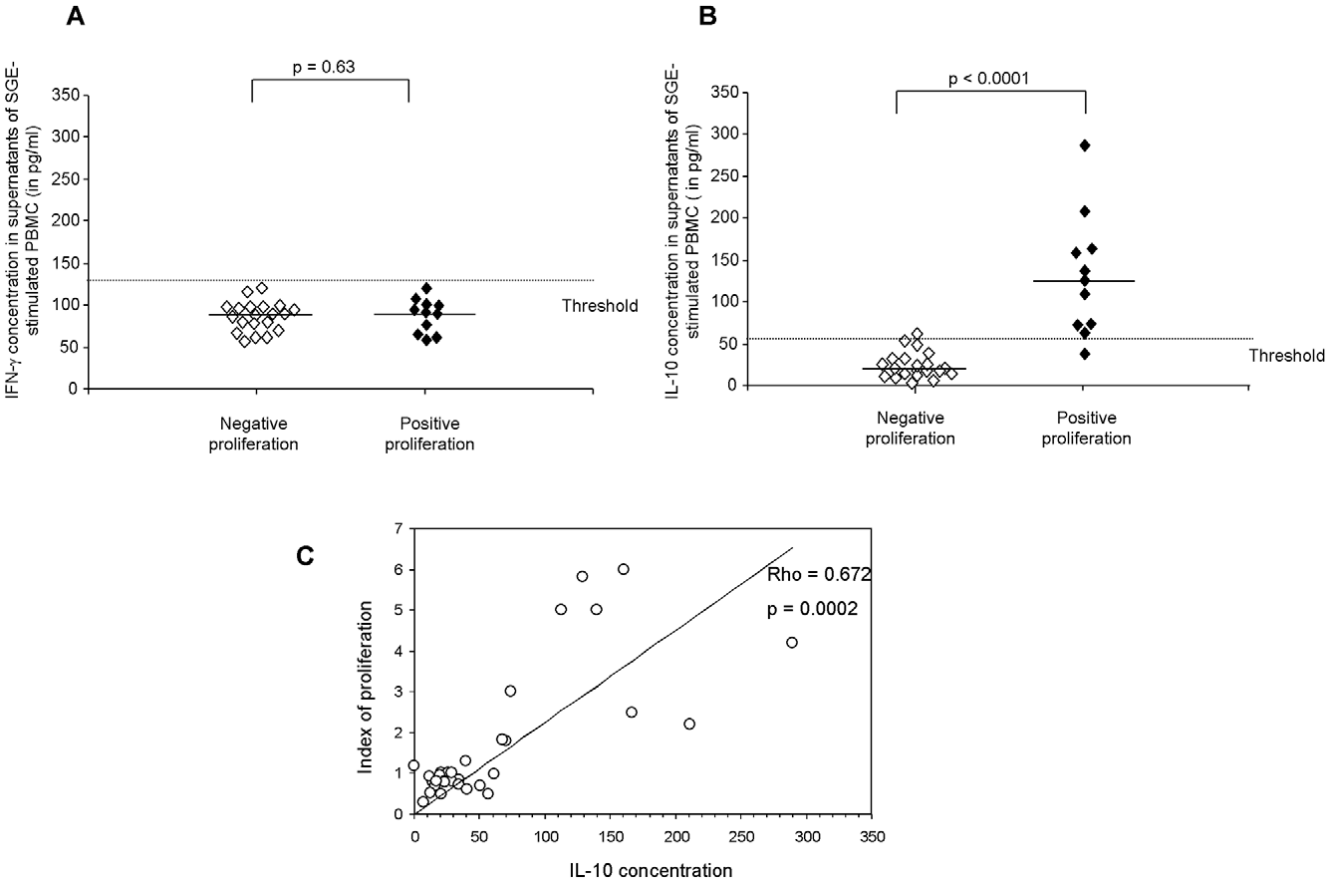
Salivary gland extracts of *Phlebotomus papatasi* induce IL-10 secretion by human PBMC in immune individuals. Purified peripheral blood mononuclear cells (0.5×10^6^ cells/mL) were stimulated with salivary gland extract of *P. papatasi.* IFN-γ (**A**) and IL-10 (**B**) secretion was evaluated in supernatants of cultures by an ELISA test at day 3 and day 2, respectively. Results were expressed as cytokine concentrations in stimulated cultures for individuals with or without PBMC proliferation against salivary gland extract. The threshold was calculated as the 95^th^ percentile of the values obtained in individuals with negative proliferation. Horizontal bars indicate median levels of cytokines. The correlation between IL-10 concentrations in supernatants of stimulated cells and proliferative responses in all tested donors was represented in (**C**).

Cellular immune responses against SGE were further tested by monitoring cytokine secretion in supernatants of stimulated PBMC. IFN-γ was detected at a median concentration of 96 pg/ml in unstimulated PBMC **(data not shown).** In supernatants of SGE-stimulated cells, concentrations of IFN-γ were not significantly different from that detected in unstimulated conditions (p>0.05) and median levels were comparable in individuals with positive and negative proliferation against SGE (median concentrations 93 pg/ml versus 90 pg/ml respectively, p = 0.63) **(** **Figure 2A** **)**. While IL-10 was detected at a median concentration of 28pg/ml in unstimulated PBMC **(data not shown)**, IL-10 concentration in supernatants of stimulated PBMC was significantly increased in individuals showing a specific proliferation against SGE (median concentration of 128 pg/ml, p<0.0001) but not individuals with negative proliferation (median concentration of 23 pg/ml) (**Figure 2B**). When the threshold of IL-10 induction was defined as the 95^th^ percentile of the values obtained in individuals with negative proliferation, we found that IL-10 was significantly induced in stimulated PBMC from all but one of the individuals showing proliferative response against SGE **(** **Figure 2B**). Conversely, in one donor (B13) with negative proliferation against SGE, IL-10 level in supernatants of SGE-stimulated PBMC was quite superior to the threshold (**Figure 2B**). Interestingly, a significant correlation was observed between proliferation and IL-10 induction in stimulated PBMC from all donors (p = 0.0002) **(** **Figure 2C** **)**. Finally, the effects of SGE on IL-10 production did not seem to be related to LPS contamination as no difference in IL-10 production by PBMC stimulated with SGE with or without polymyxin B was observed. Moreover, TNF-α was not induced after SGE stimulation.

To better define the features of the cellular population activated by the salivary gland extract, we focused our study on five representative donors (identified as B2, B6, B11, B12 and B20) from the group of donors showing proliferative response against SGE. These donors were selected as they covered the different range of proliferation positivity and were demographic matched to the remaining of the cohort (2 from the non endemic area and 3 from the endemic area). Intracytoplasmic expression of cytokines (IFN-γ, IL-10, IL-4 and TNF-α) and granzyme B was studied by flow cytometry in freshly isolated peripheral cells obtained from these donors.

**Figure 3:**
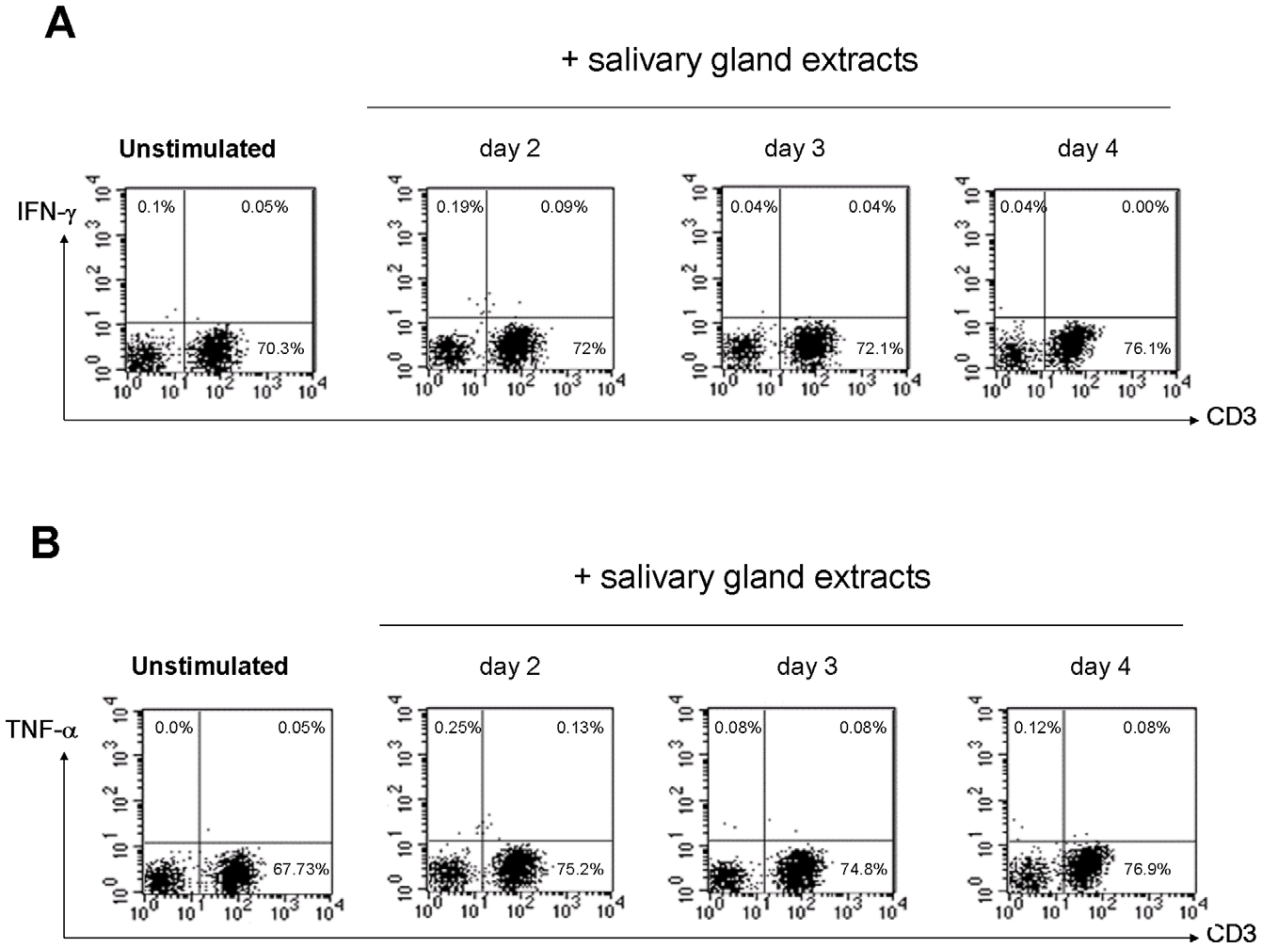
Salivary gland extracts of *Phlebotomus papatasi* did not induce IFN-γ or TNF-α production in stimulated PBMC. Freshly purified peripheral blood mononuclear cells (0.5×10^6^ cells/mL) from 5 individuals (B2, B6, B11, B12 and B20) were stimulated with salivary gland extracts of *P. papatasi* at 1 gland/ml during 96h. Intracytoplamic expression of IFN-γ (**A**) and TNF-α (**B**) was studied from days 2 through 4 by flow cytometry in CD3 membrane stained-cells. The result from individual B11 is shown.

In accordance with data obtained above, SGE did not induce IFN-γ production at any of the tested time points **(** **Figure 3A** **)**. Similar results were obtained after stimulation by phorbol myristate acetate (PMA) and ionomycin, used to increase the sensitivity of the test **(data not shown)**. Contrastingly, SLA (Soluble *Leishmania* antigen) induced in donors B2, B11, B12 and B20 (with positive proliferation against such antigen), the production of high amounts of IFN-γ **(data not shown)**.

**Figure 4:**
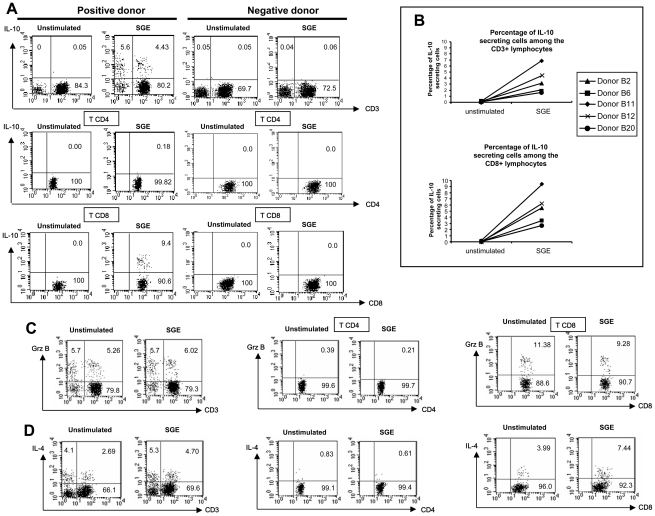
Salivary gland extracts of *Phlebotomus papatasi* activate IL-10- and IL-4-producing CD8 T lymphocytes. Freshly purified peripheral blood mononuclear cells (PBMC at 0.5×10^6^ cells/mL) from 4 (B2, B6, B11 and B12) or 5 individuals (B2, B6, B11, B12 and B20) were stimulated with salivary gland extract of *P. papatasi* at 1 gland/ml. Intracytoplamic expression of IL-10 (**A and B**), Granzyme B (**C**) and IL-4 (**D**) was studied by flow cytometry at day 3 in CD3, CD4 and CD8 membrane stained-cells. Cytokine staining was analyzed in the lymphocyte gate, on CD4+CD3+ gated cells (T CD4) or on CD8+CD3+ gated cells (T CD8). In panel A, C and D, the results from one representative donor (B11) who exhibits proliferative response to SGE are shown. Additionally, the result from the donor A3 with no proliferative response to SGE, is shown in panel A.

Similarly, TNF-α secretion was not induced by SGE (**Figure 3B**). By contrast, IL-10 was detected in 4.8% to 11% of total PBMC that have been stimulated with SGE **(** **Figure 4A** **and not shown)**. Surprisingly, IL-10 was produced primarily by CD8+ T lymphocytes and CD3-negative cells. Indeed, SGE induced the production of IL-10 in 2.4% to 9.4% (median 5%) of CD8+ T cells **(** **Figure 4A and 4B** **)**. Notably, we also performed flow cytometry analyses in two individuals (B3 and B8) living in non endemic area of leishmaniasis and who did not exhibit neither PBMC proliferation nor IL-10 production after stimulation with SGE. No cytokine production was detected (**Figure 4A** **and** **not shown**).

The detection of specific CD8+ T cells was rather unexpected. The induction of granzyme B was assessed after SGE stimulation to determine whether these cells displayed cytotoxic functions. As shown in **Figure 4C**, granzyme B was detected in almost 11% of unstimulated CD8+ T lymphocytes. The percentage of granzyme-B-producing-CD8+ cells did not increase after SGE stimulation (median percentage of 7.62% in stimulated versus 7.15% in unstimulated cells, p>0.05). This suggests that SGE did not activate cytotoxic CD8+ T lymphocytes. By contrast, the percentage of IL-4-producing CD8+ T lymphocytes increased after SGE stimulation from a mean percentage of 2.03% to 4.87% further supporting that activated CD8+ T cells are Th2 cells **(** **Figure 4D** **and not shown)**. Contrasting with results obtained with IL-10, SGE stimulation did not induce a substantial IL-4 production by CD3 negative cells **(** **Figure 4D** **)**.

### Salivary gland extract from *Phlebotomus papatasi* elicits a cellular immune response mediated by CD8+ T cells of Th2 phenotype and IFN-γ- producing CD4+ T cells

**Figure 5:**
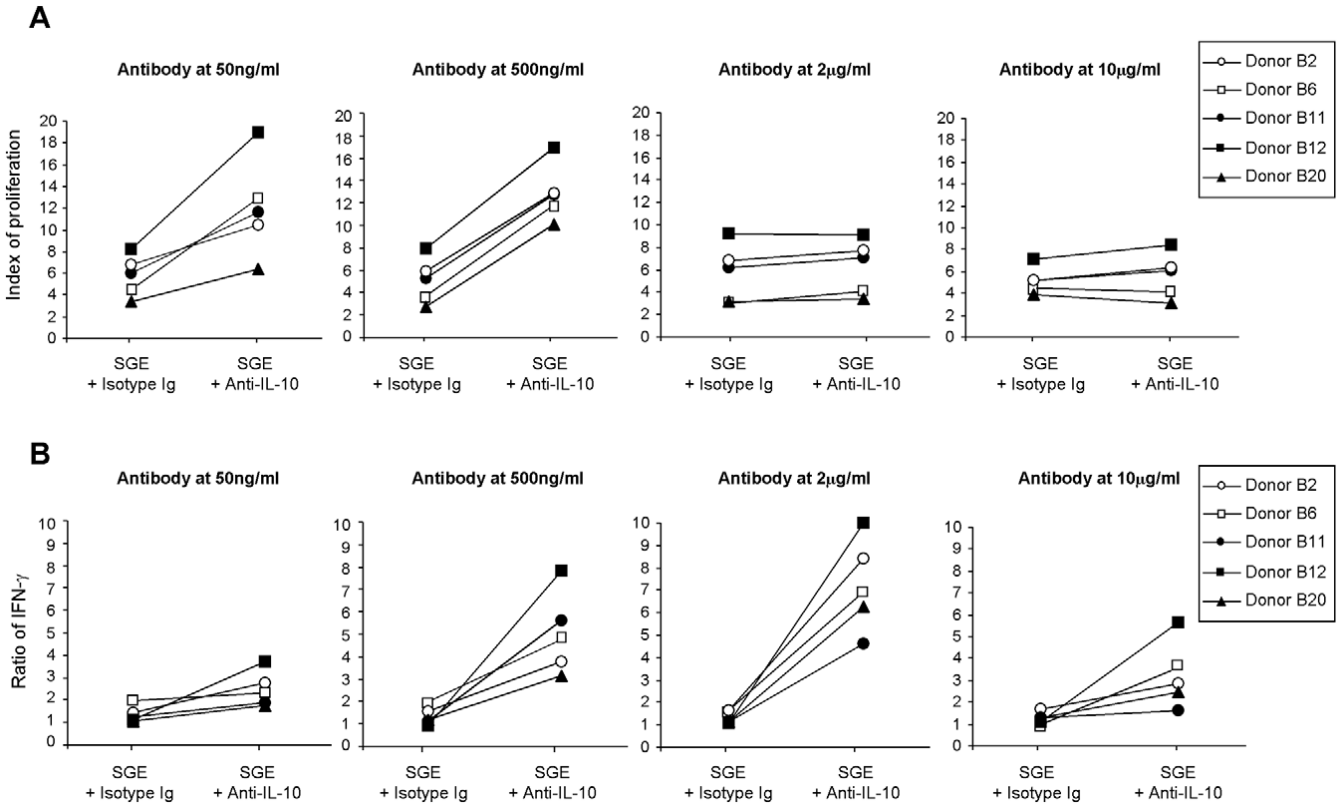
Effect of anti-IL-10 blocking antibody on lymphocytes stimulated with salivary gland extracts of *Phlebotomus papatasi*. Total PBMC (0.5×10^6^ cells/mL) from donors B2, B6, B11, B12 and B20 were stimulated with salivary gland extract (1 gland/ml) for 5 days in the presence of blocking anti-IL-10 or isotype control at the indicated concentrations. (**A**) Proliferative responses were assessed by (^3^H) thymidine uptake. Results were expressed as index of proliferation: mean counts of triplicates in antigen-stimulated cultures /mean counts of triplicates in unstimulated cultures. (**B**) IFN-γ secretion was evaluated in supernatants of cultures at day 3 using an ELISA test. Results were expressed as a ratio of cytokine levels in stimulated/unstimulated cultures.

We then tested whether the high production of IL-10 by CD8+ lymphocytes and CD3-negative cells could account for the relatively low proliferation of PBMC and the absence of IFN-γ production in response to SGE. Blocking anti-IL-10 antibody was used at different concentrations since the amount of IL-10 detected in the supernatants of stimulated lymphocytes was variable from one donor to another. As shown in Figure 5A and 5B, adding a blocking IL-10 antibody significantly enhanced the proliferation and IFN-γ production of stimulated PBMC from individuals with positive proliferation (B2, B6, B11, B12 and B20).

**Figure 6:**
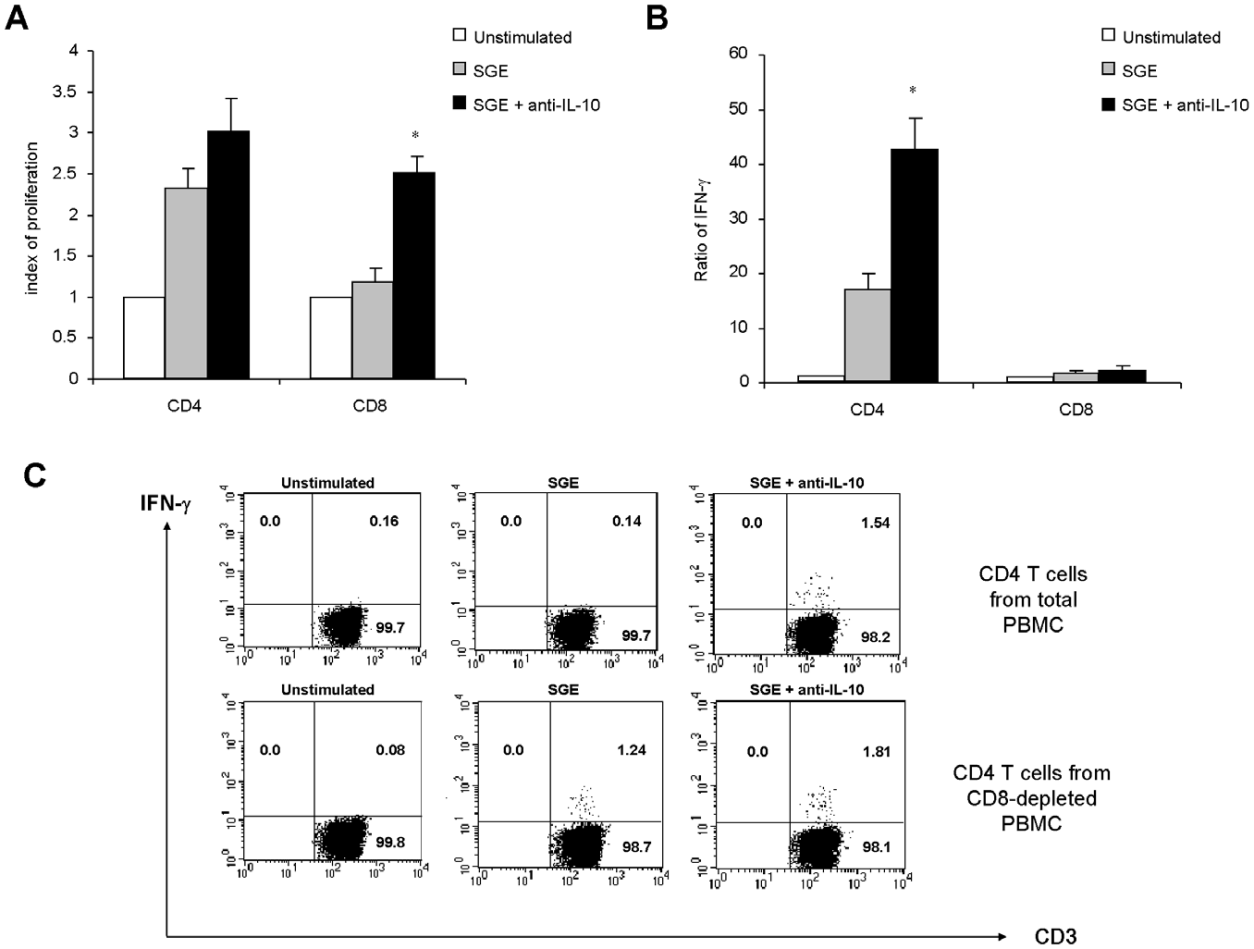
Effect of IL-10 blockage and CD8 cell depletion on lymphocytes stimulated with salivary gland extracts of *Phlebotomus papatasi*. Total peripheral blood mononuclear cells (PBMC) or PBMC depleted of CD4+ or CD8+ T lymphocytes from donors B2, B6, B11, B12 and B20 (0.5×10^6^ cells/mL) were stimulated with salivary gland extract (1 gland/ml) for 5 days in the presence or not of anti-IL-10 blocking antibody at 500ng/ml. (**A**) Proliferative responses were assessed by [^3^H] thymidine uptake. Results were expressed as index of proliferation: mean counts of triplicates in antigen-stimulated cultures /mean counts of triplicates in unstimulated cultures. (**B**) IFN-γ secretion was evaluated in supernatants of cultures at day 3 using an ELISA test. Results were expressed as a ratio of cytokine levels in stimulated/unstimulated cultures. * p<0.05 when compared to the condition with SGE. Data are representative of 5 distinct experiments. (**C**) Intracytoplamic expression of IFN-γ was studied by flow cytometry at day 3 in total (upper panels) or CD8-depleted PBMC (lower panels). Cytokine staining was analyzed in the lymphocyte gate, on CD4+CD3+ gated cells. Result from one representative individual (B11) is shown.

To better define the subsets of T cells that were activated by the saliva of *Phlebotomus papatasi*, the same experiments were subsequently performed on CD4+ and CD8+ T lymphocytes magnetically separated from PBMC of donors that exhibited proliferative responses to SGE. As shown in **Figure 6A**, SGE induces the proliferation of both CD4+ and CD8+ T lymphocytes. Blocking the production of IL-10 significantly enhanced the proliferation of CD8+ T lymphocytes. This suggests that the activated CD8+ T cells produce high amounts of IL-10 inhibiting subsequently their own proliferation. By contrast, SGE induced a considerable proliferation of CD4+ T cells that was only slightly enhanced after IL-10 blockage **(** **Figure 6A** **)**. SGE did not induce any IFN-γ production by CD8+ T lymphocytes confirming that the specific CD8+ T lymphocytes were not Th1 cells **(** **Figure 6B** **)**. However, when the PBMC was depleted from CD8+ T cells and stimulated with SGE, they produced a high amount of IFN-γ and this was further increased when IL-10 was neutralized **(** **Figure 6B** **)**. Intracytoplasmic analyses performed in donors B2, B6, B11, B12 and B20 confirmed that the neutralization of IL-10 as well as the depletion of CD8+ cells induces the production of IFN-γ by stimulated CD4+ T lymphocytes **(** **Figure 6C** **and not shown)**. The percentage of IFN-γ-producing cells within the stimulated-CD4 T cells varied from 0.93% to 1.54% when IL-10 was blocked and from 0.89% to 1.81% after CD8-depletion. Furthermore, the mean of fluorescence of IFN-γ staining of the activated-CD4 T cells increased after IL-10 blockage or CD8-depletion **(** **Figure 6C** **and not shown)**. For instance, in CD4 T cells from CD8-depleted PBMC, the mean fluorescence of IFN-γ staining increased from 21.4 to 34.7 after IL-10 blockage. Altogether, these results demonstrate that SGE induces the activation of CD8+ T cells of Th2 phenotype as well as Th1-polarized CD4+ T lymphocytes that are probably suppressed by IL-10- producing CD8+ T cells.

## Discussion

Previous studies in mice suggested that salivary components can be used to design vaccines able to control *Leishmania* infection and stressed the role of cellular immunity as correlate for protection [10]–[12]; [16], [17]. In humans, few data have been obtained on cellular immunity against sand fly saliva in volunteers experimentally exposed to the bites of uninfected *P. papatasi* [18] or *Lutzomyia longipalpis* [19]. In the present study, we analyzed the cellular immunity developed by naturally exposed individuals against *P. papatasi* sand fly saliva and demonstrated that salivary gland extract from uninfected sand flies induces the activation of IL-10 and IL-4-producing CD8+ T cells as well as IFN-γ-secreting CD4+ T lymphocytes.

Since there is no standard for the identification of individuals immunized against the saliva of *P. papatasi*, our strategy consisted of studying individuals who have high risk of contact with the vector of *L*. *major*. The studied population comprised individuals living in an endemic area of *L. major* transmission in Tunisia and thus at risk of being exposed to sand fly bites. We also included individuals who live in non endemic areas of cutaneaous leishmaniasis as the presence of *Phlebotomus papatasi* has also been reported in these areas [13]. We first assessed the proliferation and cytokine secretion of PBMC after stimulation with salivary gland extracts. A specific proliferative response of PMBC was evident in nearly 30% of donors. A similar percentage (24%) has been recently confirmed in a large number (n = 425) of individuals naturally exposed to sand fly bites (Kammoun et al. in preparation). Interestingly, the proliferative response was more frequently observed in individuals with a cellular immunity against *Leishmania*, a result that might reflect a more frequent exposure to sand fly bites in endemic areas. The positive cellular response against SGE was also reported in two out of the ten volunteers living in Tunis, corroborating the data reporting the presence of *P. papatasi* in the northern parts of Tunisia [13]. Except for the donor B7 who has been accidentally exposed to *Leishmania* antigens while manipulating parasites in the laboratory, 15 individuals exhibit positive proliferation against SLA but did not develop any cellular response to SGE. Such donors, however, developed specific antibodies against saliva as demonstrated by ELISA and/or Western blot analysis **(** **Table 1** **),** thus indicating a previous contact with the saliva of *P. papatasi*. For such donors, it seems that there was no correlation between the antibody and the cellular response. To our knowledge, the proliferative immune response toward the saliva of sand flies was not previously described in the literature although several works reported the presence of specific antibodies in people naturally exposed to sand fly bites. One possible explanation is the dominance of IL-10 production in response to the saliva of *P. papatasi*. This IL-10 will inhibit both proliferation and IFN-γ production of CD4 T cells. Furthermore, humans are outbred and therefore very diverse in term of pattern of immune response.

In contrast with results showing an increased production of IFN-γ at the inoculation site in mice sensitized with *P. papatasi* salivary proteins [10], our data did not detect any IFN-γ production by stimulated PBMC at all tested time-points. Noticeably, monitoring cytokine production by PBMC either on supernatant cell culture or using intra-cytoplasmic analysis revealed an increased synthesis of IL-10. The induction of IL-10 by sand fly saliva was previously reported in mice [20]. The production of IL-10, particularly by innate effectors such as macrophages, may explain the enhancing effect of the saliva on leishmania infectivity. Accordingly, *in vitro* experiments have demonstrated that the saliva from either *P. papatasi* or *L. longipalpis* exhibit immunosuppresive effects on macrophages and reduce the nitric oxid production [8], [9], [21]. The latter effect was ascribed to adenosine [22], an immunomodulatory component of *P. papatasi* saliva known to induce IL-10 [23]. In humans, however, little is known about the cytokine pattern activated by sand fly saliva. While the saliva of *L. longipalpis* can activate both IFN-γ and IL-10 synthesis in stimulated PBMC [19], the salivary gland lysates from *P. papatasi* inhibit the production of IFN-γ from *L. major*-stimulated PBMC [24], an effect which may be ascribed to IL-10. Interestingly, in our study, blocking IL-10 enhanced the proliferation of stimulated PBMC suggesting that IL-10 production may explain the relatively low rate of PBMC proliferation and perhaps the difficulty of detecting a proliferative response against SGE either in humans or in mice (unpublished data). Accordingly, Rohousova et al. reported an inhibitory effect of *P. papatasi* saliva on lymphocyte proliferation in mice [25]. Yet, despite the fact that IL-10 suppresses the proliferation of PBMC, we demonstrated that the production of IL-10 was mainly detected in the cells, which proliferated in response to SGE. Such paradoxical data could be easily explained. In fact, the induction of IL-10 by SGE could be illustrated only in patients who were previously exposed to *Phlebotomus papatasi* bites and so exhibiting proliferative responses to SGE. Interestingly, in one donor who exhibited a moderate production of IL-10 by stimulated PBMC, the cell proliferation was demonstrated only when the effect of IL-10 was blocked. In a current prospective work studying the cellular immune response of four hundred donors living in endemic areas of leishmaniasis in Tunisia, similar results were obtained and in approximately 10% of donors, the proliferative response of SGE-stimulated PBMC was revealed after IL-10 neutralization (Kammoun et al. manuscript in preparation). Interestingly, the five donors included in our study to define the phenotype of activated cells by SGE have been chosen as they covered the different range of proliferation against saliva and were demographic matched to the remaining donors of the cohort. Among them, four were in contact with *Leishmania* while one was not. The cellular immune response against the saliva was comparably dominated by IL-10 production whatever the donor has been in contact with the parasite or not. Indeed, the level of IL-10 in the supernatant of SGE stimulated PBMC was similar in the 5 donors **(data not shown)**. Although SLA and SGE do not activate the same effector cells, some experiments in which PBMC were co-stimulated with both antigens were performed. Adding SLA did not change the pattern of immune response against SGE that remained dominated by IL-10 **(data not shown)**.

Unexpectedly, our analysis of the activated cells revealed that CD8+ T lymphocytes were a major source of IL-10 synthesis. Detection of T CD8+ specific T cells was rather unexpected and the analysis of IL-4 expression confirmed the Th2 phenotype of these cells. This is the first account in which SGE-specific CD8+ T cells of Th2 phenotype were reported in humans. In mice, Mbow et al. reported a direct enhancing effect of *P. papatasi* saliva on IL-4 expression in the absence of *Leishmania* infection [26]. Another study revealed large numbers of IL-10 producing CD4+ and CD8+ T cells in draining lymph node of mice injected with SGE. However, since SGE was co-injected with the parasite, the direct effect of the saliva remains uncertain [20]. In donors with positive proliferation against SGE, a significant percentage of CD3 negative cells (detected on the lymphocyte gate) also produced IL-10 when stimulated with SGE. Preliminary experiments using CD14 and CD16 staining suggest that these cells were not monocytes or NK cells. B lymphocytes could be a possible cellular source of IL-10. Analysis of such hypothesis is under progress as it could bring new insights consistent with the recent literature showing the role of regulatory B lymphocytes in autoimmunity and infectious diseases [27], [28].

Compelling data suggest that the protective effect of pre-exposure to saliva in mice results from the skewing of the anti-leishmania immunity towards a Th1 protective response [10], [12]. Strikingly, our experiments showed that in donors exhibiting proliferative response against SGE, the presence of IFN-γ-producing CD4+ T lymphocytes could be revealed after neutralization of IL-10 production or depletion of CD8 lymphocytes. This indicates that the overall effect of *P. papatasi* saliva, which is dominated by the production of IL-10, may inhibit the activation of the specific Th1 cells. One may thus expect that the recall of IL-10-producing T lymphocytes in individuals previously sensitized with sand fly saliva would lead to the commitment of the immunity to *Leishmania* towards a Th2 response. This would be consistent with the lack of a protective effect of the pre-exposure to sand fly saliva in humans. Accordingly, epidemiological data indicate that the incidence of cutaneous leishmaniasis in the Old World is high in endemic areas despite the common occurrence of bites from uninfected sand flies [29]. Furthermore, recent studies indicate that the presence of antibodies against the saliva of *P. papatasi* in individuals living in endemic areas of ZCL in Tunisia was associated with an increased risk of disease [H. Louzir, personal communication, 15]. In the current prospective work mentioned above, monitoring either the cellular and humoral immune response against sand fly saliva in people living in endemic areas of cutaneous leishmaniasis throughout several transmission seasons could provide some clues regarding the effect of such immunity on the natural history of leishmaniasis. Strikingly, the effect of pre-exposure to saliva on the outcome of leishmaniasis in humans may differ from the reported effect in mice. Even in mice, conflicting results were obtained when the saliva of other species of sand flies was used [30]. Interestingly, a recent report from Rohousova et al. [31] gave an attractive explanation to the conflicting data between the possible non-protective role of sand fly bites suggested by observations from the field and the experimental data on mice. Indeed, the authors showed that the protective effect of pre-exposition to *Phlebotomus duboscqi* bites was limited to short-term exposure while a long-term exposure regimen to saliva, a scheme close to what occur in naturally exposed individuals to sand fly bites, was not protective. The authors hypothesized that an immunization with a large antigen load tends to skew the immune system towards a Th2 response, which could not be associated with protection.

Saliva is composed of a large broad of proteins. Variations in the protein composition and antigenicity of the saliva from different sand fly species may account for the conflicting results obtained from different studies [32]. Genetic differences between hosts may also underlie the disparity of the immune responses elicited by salivary proteins from the same sand fly species [17], [33] and may perhaps explain the putative differential effects of the *P. papatasi* saliva between humans and mice. Additionally, the sand fly saliva may elicit a response that differs from that elicited by its separate proteins [16]. For instance, distinct pattern of immune response could be induced by different salivary proteins from *P. papatasi* resulting in contrasting outcomes of *L. major* infection in mice [16]. PpSP15 had a protective effect, which correlated with a Th1 anti-*Leishmania* immune response whereas PpSP44 (gi|15963519) exacerbated the disease. Our data indicate that the saliva of *P. papatasi* can stimulate human CD4+ and CD8+ T cells with contrasting cytokine profiles thereby suggesting the implication of different salivary components in the activation of these populations. Defining these salivary proteins would be crucial to predict their specific effects on the outcome of leishmaniasis and to determine potential vaccine candidates.

Salivary antigens that are introduced in the host skin are drained to the lymph node where the immune response is triggered after activation of circulating lymphocytes. Reactivation of an acquired immunity against the saliva may lead to protection or exacerbation of *L. major* infection by skewing the immunity against the concomitantly inoculated *Leishmania* antigens to a Th1 or Th2 pattern, respectively. Several data obtained in mice suggested that the immunity against sand fly saliva might also act on *Leishmania* infection by creating an inhospitable *in situ* environment for the establishment of parasites. Hence, it would be critical to characterize the nature and phenotype of cells that are recruited *in situ* following exposure to bites of uninfected sand flies.

To our knowledge, this is the first account that describes the different features of the adaptive cellular immunity against the sand fly saliva in humans. When tested in the whole PBMC, salivary gland extracts from *P. papatasi* induced a low rate of proliferation, which contrasted with significant levels of IL-10 and the absence of IFN-γ. Preliminary results suggest that the CD8+ T cells activated by the saliva of *P. papatasi* could be γδ T lymphocytes and that the target components might be adenylated antigens as recently described [34], [35]. These data need however further confirmation. Whatever the target salivary component triggering the activation of IL-10 producing cells, this pattern of immune response may favor the *Leishmania* infection and facilitate the multiplication of the parasite co-injected with the saliva. Our data also demonstrated the activation of specific IFN-γ-producing CD4+ T cells that was revealed after the separation of T cell subsets. This is of great interest as it provides a new rationale for immunological approaches targeting the salivary components activating a Th1 response that could be useful for vaccination against leishmaniasis.

## Supporting Information

Figure S1 **Preliminary experiments determining the optimal condition for cell proliferation with salivary gland extracts of** ***Phlebotomus papatasi*** **.** Peripheral blood mononuclear cells (PBMC) were isolated from 3 volunteer donors living in endemic area for ZCL (B11, B12 and B13). PBMC (0.5×10^6^ cells/mL) were stimulated in triplicates in 96-well plates with salivary gland extracts at different concentrations (0.25 gland/mL, 0.5 gland/mL, 1 gland/mL and 2 gland/mL) for 3, 4 and 5 days. Proliferative responses were assessed by (^3^H) thymidine uptake. PBMC proliferation was obtained with donors B11 and B12. Results are expressed as mean of cpm (count per minute) obtained in triplicates. The optimal results (highest index of proliferation corresponding to the ratio of cpm in stimulated condition / unstimulated one) were obtained with 1 gland/mL during five days.
(TIF)
