# Supplementary figures and images for: Human Cellular Immune Response to the Saliva of Phlebotomus papatasi Is Mediated by IL-10-Producing CD8+ T Cells and Th1-Polarized CD4+ Lymphocytes
Source: PLoS Negl Trop Dis. 2011 Oct 4;5(10):e1345. doi: 10.1371/journal.pntd.0001345 (PMC3186761; doi:10.1371/journal.pntd.0001345)

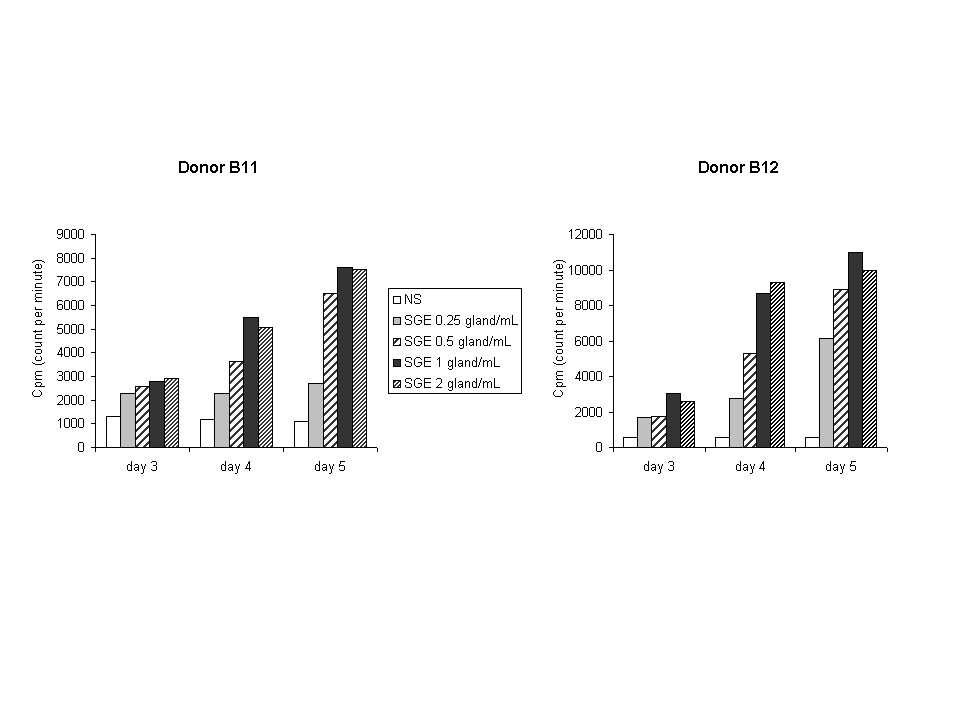

Supplement: Figure S1 — Preliminary experiments determining the optimal condition for cell proliferation with salivary gland extracts of Phlebotomus papatasi . Peripheral blood mononuclear cells (PBMC) were isolated from 3 volunteer donors living in endemic area for ZCL (B11, B12 and B13). PBMC (0.5×10^6^ cells/mL) were stimulated in triplicates in 96-well plates with salivary gland extracts at different concentrations (0.25 gland/mL, 0.5 gland/mL, 1 gland/mL and 2 gland/mL) for 3, 4 and 5 days. Proliferative responses were assessed by (3H) thymidine uptake. PBMC proliferation was obtained with donors B11 and B12. Results are expressed as mean of cpm (count per minute) obtained in triplicates. The optimal results (highest index of proliferation corresponding to the ratio of cpm in stimulated condition / unstimulated one) were obtained with 1 gland/mL during five days. (TIF) [file pntd.0001345.s001.tif]
